# Predictors of Gallstones in Patients With Metabolic Dysfunction-Associated Fatty Liver Disease (MAFLD)

**DOI:** 10.7759/cureus.85073

**Published:** 2025-05-30

**Authors:** Uzma Soomro, Ghulam Abbas, Muhammad Aslam, Sanjay Kirshan Kumar, Syed Mudassir Laeeq, Ali Hyder, Raja Taha Yaseen Khan, Nasir Hassan Luck

**Affiliations:** 1 General Surgery, Sindh Institute of Medical Sciences, Karachi, PAK; 2 Gastroenterology and Hepatology, Allama Iqbal Medical College, Lahore, PAK; 3 Gastroenterology, Madina Teaching Hospital, Faislabad, PAK; 4 Gastroenterology, Ahalia Hospital, Abu Dhabi, ARE; 5 Hepatogastroenterology, Sindh Institute of Urology and Transplantation, Karachi, PAK; 6 Gastroenterology, Chandka Medical College, Shaheed Mohtarma Benazir Bhutto Medical University, Larkana, PAK

**Keywords:** diabetes mellitus, gallstones, non-alcoholic fatty liver disease, pakistan, predictors

## Abstract

Introduction

Metabolic dysfunction-associated fatty liver disease (MAFLD) has become the most prevalent chronic liver disease worldwide, particularly in South Asian countries like Pakistan. Gallstone disease (GSD), often associated with metabolic abnormalities, shares several risk factors with MAFLD, such as obesity, diabetes, and dyslipidemia. Despite international evidence supporting an association between MAFLD and GSD, there is a hdlscarcity of local data in this regard. Therefore, this study aimed to determine the independent predictors of GSD in MAFLD patients in Pakistan.

Study methodology

This cross-sectional study was conducted at the Hepatogastroenterology Department of Sindh Institute of Urology and Transplantation (SIUT) from January to December 2024. A total of 350 adults with the presence of fatty liver on ultrasound were enrolled. Patients with significant alcohol intake, those with known liver diseases, prior cholecystectomy, pregnancy, and use of lipid-altering medications were excluded from the study. Demographic, clinical, and laboratory parameters were recorded. Gallbladder stones were identified using abdominal ultrasound. Univariate analysis followed by multivariate logistic regression was performed to identify the independent predictors of GSD.

Results

Among 350 patients with MAFLD, 102 (29.1%) had gallstones. Patients with gallstones were significantly older (mean age 52.1 vs. 47.1 years, p<0.001), with a higher prevalence of female gender (63.7% vs. 35.4%, p<0.001) and diabetes mellitus (68.6% vs. 48.3%, p = 0.002). Laboratory markers including alkaline phosphatase (ALP), gamma-glutamyl transferase (GGT), total cholesterol, triglycerides, and fasting blood sugar (FBS) were significantly elevated in the gallstone group, whereas high-density lipoprotein (HDL) was significantly lower. Multivariate logistic regression identified increasing age (OR 1.04), female gender (OR 2.73), diabetes mellitus (OR 1.89), elevated ALP (OR 1.02), low HDL (OR 1.76), and elevated FBS (OR 1.31) as independent predictors of gallstones in MAFLD.

Conclusion

In this study, 29.1% of MAFLD patients had GSD. Older age, female gender, diabetes, elevated ALP, low HDL, and high FBS were the factors independently associated with the presence of GSD in MAFLD. Further multi-centered prospective studies incorporating dietary, hormonal, and genetic factors are required for the validation of these results.

## Introduction

Metabolic dysfunction-associated fatty liver disease (MAFLD) has quickly emerged as the leading cause of chronic liver disease globally, with an estimated prevalence ranging from 25% to 30% [1,2]. MAFLD represents a spectrum ranging from simple steatosis to steatohepatitis (MASH), fibrosis, and cirrhosis [3,4]. South Asian populations like Pakistan are facing an alarming surge in MAFLD due to sedentary lifestyles accompanied by poor dietary patterns and an obesity and diabetes epidemic. A systematic review from Pakistan estimated the prevalence of MAFLD to be around 14%-34% among the general population, with increased rates among type 2 diabetes and metabolic syndrome patients [5].

Gallstone disease (GSD) represents another important disorder of the hepatobiliary system that is defined by the presence of calculi in the gallbladder [6]. It particularly affects individuals with metabolic abnormalities [7]. Globally, gallstones are detected in approximately 10%-20% of adults, and interestingly, the prevalence overlaps remarkably with risk factors of MAFLD, including obesity, insulin resistance, dyslipidemia, and metabolic syndrome [6,7]. Because these risk factors are so shared by both MAFLD and GSD, researchers hypothesized an underlying pathophysiologic connection linking MAFLD and gallstone formation [8,9]. This association has been examined in several international studies. Fracanzani et al. (2012) showed that MAFLD patients had an increased rate of gallstone formation [10]. Similarly, an American study by Ruhl et al. revealed that patients with MAFLD had an increased risk of GSD as compared to the normal population [11]. Asian evidence further substantiated these findings through a study in the Chinese population that showed the association between hepatic steatosis and the risk of gallstones [12].

In Pakistan, data are scarce regarding not only the prevalence of gallstones in the MAFLD population but also the factors predicting GSD in this population. In one cross-sectional study from Pakistan, 24.2% of patients with MAFLD were found to have associated gallstones; however, the predictors of GSD in this population are not well described [8]. Identification of predictors of GSD in MAFLD patients, particularly in the Pakistani population, is of great clinical relevance. Early detection will be helpful in risk stratification, screening, and early intervention to avoid complications like cholecystitis, biliary colic, or gallstone pancreatitis. Therefore, the aim of this study was to identify predictors of GSD in the Pakistani population with MAFLD.

## Materials and methods

This cross-sectional study was carried out at the Department of Hepatogastroenterology, Sindh Institute of Urology and Transplantation (SIUT) from January 2024 to December 2024 after approval from the ethical review committee (ERC, SIUT) (A-1163). After obtaining informed consent, all adult patients aged ≥18 years with the presence of fatty liver on ultrasound were enrolled in the study. However, patients with a history of significant alcohol consumption (>20 g/day for women, >30 g/day for men), those with a history of liver disease (e.g. viral hepatitis, autoimmune hepatitis, and hemochromatosis), patients with prior history of cholecystectomy, pregnant females, and patients on medications known to influence the lipid metabolism (e.g. statins and fibrates) were excluded from the study.

Using a reported prevalence of GSD in MAFLD patients of approximately 24% (based on regional data), with a 95% confidence interval and 5% margin of error, the calculated sample size was 310 patients. To account for incomplete data, a total of 350 NAFLD patients were enrolled.

Data collection

After taking the consent, demographic data, clinical history, and laboratory parameters such as hemoglobin (Hb), total leukocyte count (TLC), platelet count, serum bilirubin, alanine aminotransferase (ALT), aspartate aminotransferase (AST), alkaline phosphatase (ALP), gamma-glutamyl transferase (GGT), total cholesterol, high-density lipoprotein (HDL), low-density lipoprotein (LDL), serum triglycerides, and fasting blood sugar (FBS) were recorded in each patient at the time of enrollment. All patients then underwent abdominal ultrasonography performed by an experienced radiologist (more than 5 years of experience) to confirm the presence of fatty liver (presence of hyper-echoic liver with hepatorenal index >1 indicating steatosis) and gallbladder stones.

Data analysis procedure

The data analysis was performed using SPSS version 26.0 (IBM Corp., Armonk, NY, USA). Continuous variables were expressed as mean SD, while categorical variables were expressed as frequencies and percentages. Continuous variables were analyzed using the student t-test while the Chi-square test was used for the analysis of categorical variables. The outcome was recorded in terms of the presence or absence of gall stones on ultrasound. Statistically significant variables on univariate analysis then subsequently underwent multivariate logistic regression analysis to identify the predictors of GSD in the MAFLD population. A p-value of less than 0.05 was considered statistically significant.

## Results

A total of 350 patients diagnosed with MAFLD were enrolled in this study. Among them, 198 (56.6%) were males and 152 (43.4%) were females. The mean age of the participants was 48.7±12.5 years. In terms of presenting complaints, 252 (72%) patients reported abdominal discomfort, 189 (54%) patients had dyspepsia, and nausea and vomiting were noted in 112 (32%) patients. Diabetes mellitus was present in 189 (54%) patients, while 166 (47.4%) patients were hypertensive at the time of presentation.

Laboratory parameters across the cohort revealed a mean Hb level of 12.8±1.7 g/dL and a mean TLC of 7.9±2.1×10⁹/L. The mean platelet count was 235±56×10⁹/L. Regarding liver function tests, the mean serum bilirubin was 0.9±0.4 mg/dL, ALT was elevated at 58.3±21.6 U/L, and AST was 42.7±19.4 U/L. ALP and GGT levels were elevated as well, with means of 123.4±35.7 U/L and 78.6±28.9 U/L, respectively. Lipid profiles showed a mean total cholesterol of 212.4±39.6 mg/dL, HDL of 38.2±9.5 mg/dL, LDL of 128.3±33.1 mg/dL, and triglycerides of 189.6±54.2 mg/dL. The mean FBS level was 118.7±36.4 mg/dL. Out of the total 350 patients, 102 (29.1%) were found to have gallstones on ultrasonography (Table 1).

**Table 1:** Baseline characteristics of the studied population (n=350) TLC: total leukocyte count; ALT: alanine transaminase; AST: aspartate transaminase; GGT: gamma-glutamyl transpeptidase; LDL: low-density lipoprotein; HDL: high-density lipoprotein; FBS: fasting blood sugar; Hb: hemoglobin; ALP: alkaline phosphatase Reference ranges: Hb (g/dL): 14-16 for Men and 12-14 for women; TLC (x10^9^/L): 4-11; PLT (x10^9^/L): 250-400; total bilirubin (mg/dL): 0.9-1.2; AST (U/L): up to 40; ALT (U/L): up to 33 for men and 25 for women; ALP (U/L):105-135; GGT (U/L): up to 50; total cholesterol (mg/dL): less than 200; LDL (mg/dL): less than 110; HDL (mg/dL): 40-60; serum triglycerides (mg/dL): less than 250; and FBS: less than 90 mg/dL.

Variables		n (%)
Gender	Male	198 (56.6)
	Female	152 (43.4)
Presenting symptoms	Abdominal discomfort	252 (72)
	Dyspepsia	189 (54)
	Nausea and vomiting	112 (32)
	Asymptomatic	63 (18)
Comorbidities	Hypertension	166 (47.4)
	Diabetes	189 (54)
Variable		Mean±SD
Age (years)		48.7±12.5
Hb (g/dL)		12.8±1.7
TLC (x10^9^/L)		7.9±2.1
Platelet count (x10^9^/L)		235±56
Total Bilirubin (mg/dL)		0.9±0.4
ALT (IU/L)		48.3±21.6
AST (IU/L)		42.7±19.4
GGT (IU/L)		78.6±28.9
ALP (IU/L)		123.4±35.7
Total cholesterol (mg/dL)		212.4±39.6
Serum triglyceride levels (mg/dL)		189.6±54.2
HDL (mg/dL)		38.2±9.5
LDL (mg/dL)		128.3±33.1
FBS (mg/dL)		118.7±36.4

On comparative analysis, patients with gallstones were notably older, with a mean age of 52.1±11.8 years compared to 47.1±12.3 years in those without gallstones (p<0.001). The female predominance was significant among the gallstone group, with 63.7% being female, whereas females constituted only 35.4% of the group without gallstones (p<0.001). Diabetes mellitus was also more prevalent among the gallstone cohort (68.6% vs. 48.3%; p=0.002) (Table 2).

**Table 2:** Comparison of variables between MAFLD patients with and without gallstones (n=350) ^*^The Chi-square test was used for the calculation of p-values of categorical variables. ^**^t-test was used for the calculation of p-values of continuous variables. TLC: total leukocyte count; ALT: alanine transaminase; AST: aspartate transaminase; GGT: gamma glutamyl transpeptidase; LDL: low-density Lipoprotein; HDL: high-density lipoprotein; FBS: fasting blood sugar; Hb: hemoglobin; MAFLD: metabolic dysfunction-associated fatty liver disease Reference ranges: Hb (g/dL): 14-16 for men and 12-14 for women; TLC (x10^9^/L): 4-11; PLT (x10^9^/L):250-400; total bilirubin (mg/dL): 0.9-1.2; AST (U/L): up to 40; ALT (U/L): up to 33 for men and 25 for women; GGT (U/L): up to 50; total cholesterol (mg/dL): less than 200; LDL (mg/dL): less than 110; HDL (mg/dL): 40-60; serum triglycerides (mg/dL): less than 250; and FBS: less than 90 mg/dL

Variable	MAFLD with gallstones (n=102) N (%)	MAFLD without gallstones (n=248) N (%)	Chi-square (χ²) value^*^/ t-test value^**^	P-value
Female gender	97 (63.9)	55 (36.1)	χ²=24.6	<0.001
Diabetes mellitus	130 (69)	59 (31)	χ²=10.1	0.002
Hypertension	79 (47.6)	87 (52.4)	χ²=0.371	0.174
Age (years)	52.1±11.8	47.1±12.3	t=3.68	<0.001
Hb (g/dL)	12.4±1.6	13.0±1.7	t=-3.32	0.59
TLC (x10^9^/L)	7.6±2.1	7.9±2.2	t=0.968	0.14
PLT (x10^9^/L)	245±81	221±76	t=0.714	0.749
ALT (U/L)	52.6±22.4	46.3±20.8	t=1.44	0.045
AST (U/L)	45.8±18.9	43.3±19.6	t=1.03	0.091
ALP (U/L)	134.2±36.1	118.7±34.9	t=3.81	<0.001
GGT (U/L)	84.7±29.7	75.7±27.9	t=2.89	0.004
Total cholesterol (mg/dL)	220.8±40.7	208.3±38.2	t=2.67	0.008
HDL (mg/dL)	36.4±8.7	39.1±9.8	t=-2.63	0.009
LDL (mg/dL)	132.7±32.8	126.4±33.3	t=1.79	0.073
Triglycerides (mg/dL)	198.3±53.9	185.7±54.4	t=2.09	0.037
FBS (mg/dL)	126.9±38.7	115.3±35.1	t=3.14	0.002

Laboratory comparisons between patients with and without gallstones revealed significant differences. Liver enzymes were elevated in patients with gallstones, with ALT at 52.6±22.4 U/L compared to 46.3±20.8 U/L in those without stones (p=0.045). ALP levels were also notably higher in the gallstone group (134.2±36.1 U/L vs. 118.7±34.9 U/L; p<0.001), as were GGT levels (84.7±29.7 U/L vs. 75.7±27.9 U/L; p=0.004) (Table 2).

Regarding lipid parameters, total cholesterol was significantly elevated in patients with gallstones (220.8±40.7 mg/dL vs. 208.3±38.2 mg/dL; p=0.008). HDL levels were lower in the gallstone group (36.4±8.7 mg/dL) compared to the non-gallstone group (39.1±9.8 mg/dL; p=0.009), while triglyceride levels were higher among those with gallstones (198.3±53.9 mg/dL vs. 185.7±54.4 mg/dL; p=0.037). FBS levels were also elevated in the gallstone group (126.9±38.7 mg/dL vs. 115.3±35.1 mg/dL; p=0.002) (Table 2).

On multivariate logistic regression analysis, increasing age (OR 1.04 per year increase; 95% CI: 1.02-1.07; p<0.001), female gender (OR 2.73; 95% CI: 1.75-4.26; p<0.001), presence of diabetes mellitus (OR 1.89; 95% CI: 1.21-2.96; p=0.005), raised ALP (OR 1.02 per unit increase; 95% CI: 1.01-1.03; p<0.001), decreased HDL levels (OR 1.76 per mg/dL decrease; 95% CI: 1.62-2.21; p=0.003), and elevated FBS levels (OR 1.31 per mg/dL increase; 95% CI: 1.10-1.47; p=0.013) were the factors independently associated with the presence of GSD in patient with MAFLD (Table 3).

**Table 3:** Multivariate analysis showing independent predictors of gallstones in MAFLD ALT: alanine transaminase; LDL: low-density lipoprotein; HDL: high-density lipoprotein; FBS: fasting blood sugar; MAFLD: metabolic dysfunction-associated fatty liver disease; ALP: alkaline phosphatase; AOR: adjusted odds ratio

Variable	AOR	95% CI	P-value
Age (per year increase)	1.04	1.02-1.07	<0.001
Female gender	2.73	1.75-4.26	<0.001
Diabetes mellitus	1.89	1.21-2.96	0.005
ALP (per unit increase)	1.02	1.01-1.03	<0.001
ALT (per unit increase)	0.91	0.89-1.02	0.074
HDL (per mg/dL decrease)	1.76	1.62-2.21	0.003
Triglycerides (per mg/dL increase)	1.12	0.79-1.31	0.189
LDL (per mg/dL increase)	1.13	0.93-1.37	0.237
Total Cholesterol (per mg/dL increase)	1.11	0.81-1.22	0.264
FBS (per mg/dL increase)	1.31	1.10-1.47	0.013

## Discussion

The purpose of this study was to evaluate the clinical, biochemical, and demographic indicators of GSD in the context of a Pakistani population of patients with MAFLD. Our study found that older age, female gender, presence of diabetes mellitus, elevated ALP, low HDL, and increased FBS levels at the time of presentation were the factors that independently correlated with the presence of gallstones in the MAFLD population. This outcome supports the growing literature indicating that hepatobiliary and metabolic conditions are strongly interlinked through similar proposed pathogenic pathways.

Our analysis revealed a prevalence of gallstones in the MAFLD group of 29.1%, which was greater than the general population prevalence of 10%-20% but comparable to the results of the previous studies revealing enhanced gallstone formation in the presence of metabolic syndrome or hepatic steatosis. Koller et al. documented a higher prevalence of gallstones of 47% in the group of MAFLD cases compared to the general population rate of 26% [13]. Likewise, Ruhl et al. showed that the prevalence of GSD was doubled in the presence of hepatic steatosis (34.4% vs. 19.9%); thus, supporting our observation of increased prevalence of GSD in patients with MAFLD [11].

Female gender was also a significant predictor of gallstones in our cohort (AOR: 2.73, p<0.001). This is supported globally by the fact that women have an increased risk of GSD due to the hormonal effects, more so because of the hormone estrogen, which promotes cholesterol in bile to become saturated [14]. Another independent risk factor was increasing age, with an adjusted odds ratio (AOR) of 1.04 per year (p<0.001). The pathogenesis of gallstone disease is strongly associated with increasing age due to cumulative exposure to metabolic injury and changes in gallbladder contractility and bile composition [15]. This corresponds with findings from other large-scale studies showing an increased prevalence of gallstones in older age groups [16].

Our analysis also emphasized the role of diabetes mellitus as a major risk factor (AOR: 1.89, p=0.005), consistent with the established pathophysiologic mechanisms. Insulin resistance, the central defect of both diabetes and MAFLD, augments hepatic cholesterol production and inhibits bile acid secretion, facilitating the development of gallstones [17]. Ratheesh R et al. reported an increased risk of gallstones in diabetics, particularly in those with poor control of glycemia [18].

Among the markers of liver function, ALP was strongly correlated with gallstones (AOR: 1.02 per unit increase; p<0.001), whereas ALT and AST, while raised in the setting of gallstones, were not predictive in multivariate analysis by themselves. Raised ALP is a measure of cholestatic activity and potentially an early warning of a subclinical biliary block or dysfunction of the gallbladder in the context of MAFLD and deserves further exploration [19,20].

For lipid parameters, low HDL was the only robust predictor of gallstones (AOR: 1.76 per decrease of mg/dL; p=0.003), and neither total cholesterol, LDL, nor triglycerides remained significant in the adjusted analysis. This emphasizes the protective role of HDL not only as a cardioprotective lipid fraction but also in biliary cholesterol solubility. Zhang M et al. also showed similar results in their study, in which low HDL was directly associated with a higher incidence of gallstones, particularly in the presence of coexistent hepatic steatosis [9,20].

Our study has a number of strengths including a high sample size and robust biochemical profiling. However, there are some limitations that need to be mentioned. First, this is a single-center study, and results might not apply to other ethnic and population groups. Secondly, the cross-sectional design of the study may limit its causal inference. 

## Conclusions

Our study showed a prevalence rate of 29.1% of gallbladder stones among MAFLD patients. We observed that increasing age, female gender, presence of diabetes mellitus, elevated ALP levels, low HDL cholesterol, and elevated FBS were independent predictors of GSD in MAFLD. Identification of these predictors offers valuable clinical insights for early screening and risk stratification in MAFLD patients, potentially guiding preventive strategies and timely interventions to manage gallstone-related complications. However, for further validation of our results, there is a requirement for large data-based multicenter prospective studies evaluating dietary factors, hormonal influences, and genetic factors responsible for GSD in MAFLD.
